# Erythema annulare centrifugum associated with chronic amitriptyline intake^☆^^☆☆^

**DOI:** 10.1016/j.abd.2020.05.013

**Published:** 2020-11-19

**Authors:** Diego Fernandez-Nieto, Daniel Ortega-Quijano, Juan Jimenez-Cauhe, Sonia Bea-Ardebol

**Affiliations:** Department of Dermatology, Hospital Universitario Ramón y Cajal, Madrid, Spain

*Dear Editor,*

A 41-year-old woman presented to the hospital with a mildly pruritic exanthem which had appeared two months before. She had been presenting similar episodes for the past five years, treated with topical corticosteroids and short courses of methylprednisolone. Each episode lasted longer and was more widespread than the previous one. She denied fever or any systemic symptoms. The patient reported a history of migraines, treated with amitriptyline for the past five years and occasional anti-inflammatories. Amitriptyline was started two weeks before the first appearance of skin lesions, but the patient did not associate both events. Physical examination revealed annular and polycyclic plaques, with a trailing scale and central clearing, predominantly in lower limbs (Fig. 1). A skin biopsy from the edge of a lesion was performed, showing mild papillary edema, spongiosis, lymphocyte exocytosis and a perivascular lymphohistiocytic infiltrate in a “coat sleeve” appearance (Fig. 2). Periodic acid-Schiff staining did not show fungal forms. Fungal culture was negative. Laboratory tests including complete blood count, liver and kidney function tests, serological tests for HBV, HCV, HIVH, borrelia and syphilis, ANA, ASLO titer, rheumatoid factor, complement, IgE levels, proteinogram, β-2 microglobulin, and thyroid function test were normal. Chest radiograph, Mantoux skin test, and abdominopelvic ultrasonography were unremarkable. These findings were consistent with erythema annulare centrifugum (EAC), superficial type.

**Figure 1:**
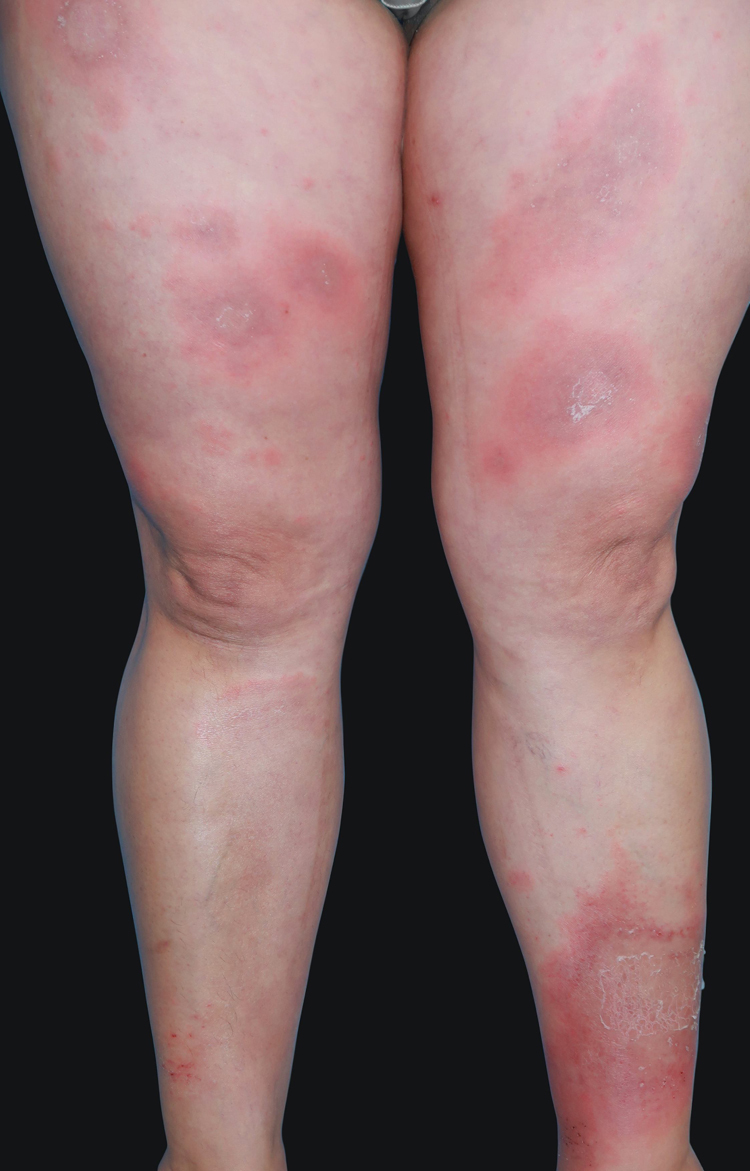
Annular erythematous plaques with trailing scale located at thighs and legs.

**Figure 2:**
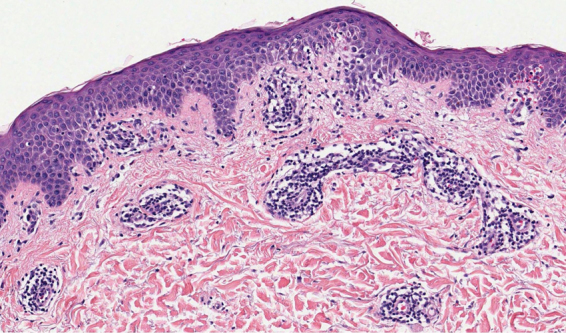
Mild spongiosis, lymphocyte exocytosis, papillary edema, and a perivascular lymphohistiocytic infiltrate in a “coat sleeve” appearance. No eosinophils were observed (Hematoxylin & eosin, ×100).

Administration of amitriptyline was suspended and mometasone furoate 0.1% cream was prescribed, showing moderate improvement at the one-month follow-up visit. Fluconazole 100 mg/day was prescribed for four weeks. Due to inefficacy, it was changed to erythromycin 250 mg four times a day for four weeks. After this treatment, the patient showed nearly complete response. At the one-year follow-up, some minor recurrences were noted, which only required short courses of topical corticosteroids. Amitriptyline oral rechallenge was refused by the patient.

EAC is classified as a reactive erythema, along with erythema chronicum migrans, erythema marginatum, and erythema gyratum repens. Each entity is separated by clinical and histopathologic correlation. EAC is divided in superficial and deep forms.^1^ The superficial form often has scaly borders tending to form on the trailing edge of the annular lesion. The deep form has non-scaly indurated borders without marked epidermal changes. The superficial type is associated with recurrences and a shorter duration of skin lesions when compared with the deep type.^1^ Common differential diagnosis includes other annular erythemas such as erythema chronicum migrans, mycosis fungoides, urticaria, psoriasis, tinea corporis, and annular sarcoidosis. Histopathology shows a lymphohistiocytic perivascular infiltrate in both superficial and deep types of EAC. In the superficial type, a perivascular infiltrate and dermal edema are located in the upper dermis. Epidermal changes such as acanthosis, spongiosis and even vesiculation can be seen. In the deep type, the perivascular infiltrate is found in the middle and lower dermis, with a “coat sleeve-like” appearance.^1^ Edema and epidermal changes are uncommon.

EAC is thought to represent a cutaneous manifestation of a type IV hypersensitivity reaction to several etiologies; however, several cases are idiopathic. Treatment and eradication of the underlying disease, if present, is usually effective. EAC has been associated with bacterial, parasitic, viral and fungal infections. Endocrine and immunological disorders such as Graves’ disease, Hashimoto thyroiditis, and Sjögren syndrome have been reported.^2^ When it occurs in a paraneoplastic setting, it usually precedes (46% of cases) or is simultaneous within one-month (33% of cases) of the discovery of the related cancer.^3^ EAC usually resolves after cancer treatment, and recurrence is associated with tumor relapse.^3^ EAC has also been related to drugs, including hydroxychloroquine, hydrochlorothiazide, spironolactone, cimetidine, salicylates, piroxicam, penicillin, ustekinumab, and amitriptyline.^2^

Amitriptyline has been classically considered a typical cause of EAC, since its proven association in 1999 by García-Doval et al.^4^ However, this is the only case reported in medical literature. In the present case, amitriptyline was suspected to be the cause of EAC, due to its temporal association and previous report. However, drug discontinuation did not resolve EAC. The chronicity of this drug intake (five years) could have triggered a perpetuated immune response that remained even after amitriptyline discontinuation. Another possible explanation is that amitriptyline was not related to EAC, and it was rather idiopathic or associated to a hidden bacterial focus. Erythromycin and azithromycin have been reported as a safe and effective therapy for EAC, as in the present case.^5^ These antibiotics may have effect on a hidden bacterial focus or play a role, due to their anti-inflammatory effect.

In conclusion, chronic drug related EAC may persist even after drug discontinuation. Macrolides are a safe and effective therapy for EAC.

## Financial support

None declared.

## Authors' contributions

Diego Fernandez-Nieto: Approval of the final version of the manuscript; design and planning of the study; drafting and editing of the manuscript; collection, analysis, and interpretation of data; effective participation in research orientation; critical review of the literature; critical review of the manuscript.

Daniel Ortega-Quijano: Approval of the final version of the manuscript; intellectual participation in the propaedeutic and/or therapeutic conduct of the studied cases; critical review of the literature; critical review of the manuscript.

Juan Jimenez-Cauhe: Approval of the final version of the manuscript; critical review of the literature; critical review of the manuscript.

Sonia Bea-Ardebol: Approval of the final version of the manuscript; effective participation in research orientation; intellectual participation in the propaedeutic and/or therapeutic conduct of the studied cases; critical review of the manuscript.

## Conflicts of interest

None declared.
